# Rapid and Specific Drug Quality Testing Assay for Artemisinin and Its Derivatives Using a Luminescent Reaction and Novel Microfluidic Technology

**DOI:** 10.4269/ajtmh.14-0392

**Published:** 2015-06-03

**Authors:** Nga T. Ho, Darash Desai, Muhammad H. Zaman

**Affiliations:** Department of Biomedical Engineering, Boston University, Boston, Massachusetts

## Abstract

Globally, it is estimated that about 10–30% of pharmaceuticals are of poor quality. Poor-quality drugs lead to long-term drug resistance, create morbidity, and strain the financial structure of the health system. The current technologies for substandard drug detection either are too expensive for low-resource regions or only provide qualitative results. To address the current limitations with point-of-care technologies, we have developed an affordable and robust assay to quantify the amount of active pharmaceutical ingredients (APIs) to test product quality. Our novel assay consists of two parts: detection reagent (probe) and a microfluidic testing platform. As antimalarials are of high importance in the global fight against malaria and are often substandard, they are chosen as the model to validate our assay. As a proof-of-concept, we have tested the assay with artesunate pure and substandard samples (Arsuamoon tablets) from Africa and compared with the conventional 96-well plate with spectrophotometer to demonstrate the quantitative efficacy and performance of our system.

## Introduction

The World Health Organization (WHO) estimates that about 10–30% of pharmaceuticals in the world are of poor quality, counterfeit, falsified or broadly speaking, substandard.^1^ From a public health perspective, a key contributor to the development and proliferation of drug-resistant strains of infections, including tuberculosis (TB), malaria, and other infections that are leading killers in resource-limited settings, is poor-quality medicines.^2^ Poor-quality and substandard medicines also create morbidity and increase mortality rates of many diseases, where early and correct treatment is crucial for saving lives. Given the pervasive nature of the problem and its substantial impact on health systems, there is a dire need for technologies and solutions to address this problem comprehensively.

There are three broad classes or methods used for the detection of substandard pharmaceuticals: high-performance liquid chromatography (HPLC), handheld Raman spectroscopy (RS) or near-infrared spectroscopy instruments, and thin-layer chromatography (TLC)-based systems. In resource-rich settings, mass spectrometer is also used for quantification of the pill ingredients. Currently, HPLC is the gold standard method used for the analysis of different active pharmaceutical ingredients (APIs) and impurities within the drug tablets.^3^ HPLC is a powerful tool for quantitative analysis of pharmaceuticals and its impurities. However, the system requires a precise pump, a good UV detector, and a laser source for some chemical compounds that are autofluorescent at certain excitation wavelength. That makes a complete system's cost too expensive (often upwards of $100,000) for low-resources settings and field testing.^4^ Moreover, appropriate maintenance and storage for the advanced devices are also required. Also, trained personnel are required for maintenance and operation of HPLC.

RS is another common method used to quantify substandard medicine. In Raman, the sample is exposed to laser light. The interaction of the laser light with molecules on the surface of the samples shift the laser's photon energy up and down and result in different scattered shifted laser light pattern called unique Raman spectra for specific chemical molecules.^5^ The advantage of RS is that it provides a noninvasive way to identify different pharmaceuticals in the tablet since no sample preparation is required. The analysis time is relatively short (10–15 minutes). The operation requires little training. However, there are several field-based challenges. The instrument itself is prohibitively expensive ($30,000–$60,000) due to the laser source.^6^ In addition, the technology only looks at the surface of the tablet and not the entire composition. Thus, if the tablet is not uniformly distributed, the quantification could be incorrect and the results misleading. Samples may decompose/degrade upon the continuous exposure to laser light for more than 10 minutes. If the molecule is autofluorescent, the emission spectrum can overlay with the Raman spectra as well.^7^

TLC is often used as a pharmaceutical analytical method in resource-limited areas. In some ways, it is a simplified version of an HPLC. A sheet of glass, aluminum, or plastic is coated with absorbent materials such as cellulose or silica gel and acts as the column in HPLC to separate the different components in nonvolatile mixtures. For visualization or detection, different dyes are spotted on the sheet to identify different chemical compounds in the mixture.^8^ One of the most popular TLC is the Minilab from Global Pharma Health Fund (GPHF). The Minilab system has been developed to work in resource-limited settings. It includes two suitcases that weigh about 100 pounds each and contain all the equipment and materials for TLC.^9^–^11^ This is recommended by WHO for the detection of fake drugs in resource-limited regions. With this method, quick, inexpensive, and qualitative results can be obtained in about a couple of hours. However, qualitative measurements cannot determine substandard drugs. The entire system is too heavy to carry to the field. The method is cumbersome and laborious. In addition, user-induced errors, due to no automation, are also high.

The current methods discussed above reveal a technology gap that can be filled with a technology that is affordable, automated, easy to use, precise, and quantitative. Our microfluidic system, combined with specific chemiluminescent reaction for drug quantification, is aimed at addressing this gap. In this paper, we introduce a simple microfluidic system that can quantify the concentration of API in a robust, reliable, quantitative, and automated system to address the challenges associated with detection of substandard medicines.

Our focus for this study is on simple antimalarials as a model system. Historically, the main drugs for malaria treatment were quinine and chloroquine. However, there have been multiple cases reported for the drug resistance with quinine and chloroquine.^12^,^13^ In response, the WHO now recommends that patients with malaria should take the artemisinin combination therapy (ACT).^14^ Unfortunately, recently, there have been many reports of artemisinin resistance in southeast Asia. One of the main causes of resistance is the use of poor-quality drugs.^15^,^16^ It has been reported that about 38–52% of artesunate (ATS) and 55% of dihydroartemisin (DHA) fail the drug quality control tests in the southeast Asian and African countries.^17^–^19^ To respond to the counterfeit and substandard ATS, currently methods are developed to detect ATS in the tablets such as desorption electrospray ionization mass spectroscopy (DESI MS) and colorimetric field tests using diazonium salts.^20^–^22^ However, both DESI and the colorimetric test are only good for qualitative measurements. For quantitative purpose, extra sample preparation and detection steps are required. As a result, quantitative and easy to use assays for the detection of artemisinin (ART) and its derivatives are in urgent need. Thus, as a model system, we chose ART and its derivatives to test, validate, and optimize our system.

## Materials and Methods

### ART chemistry.

**Figure 1.**
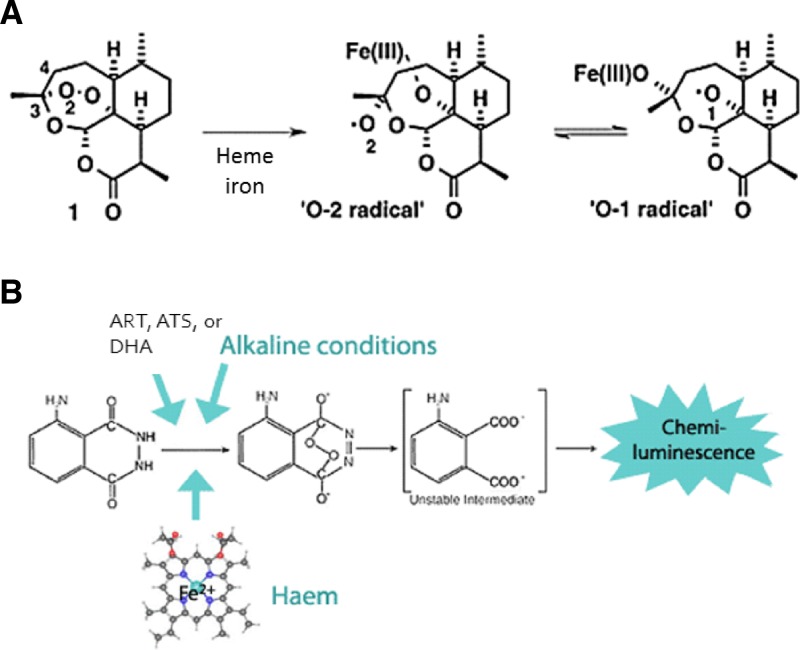
(**A**) The cleavage of the peroxide group into oxygen radicals of artemisinin. (**B**) The mechanism of the luminol reaction. Luminol in the presence of ferrous iron in heme or hematin in blood and basic environment reacts with peroxide group to transform into a new compound that emits luminescent signal at 425 nm.^23^,^24^

To develop and optimize the probe for the technology, we studied the mechanism of action of ART and its derivatives. The chemical structures of ART and its derivatives all have special peroxide groups. In the presence of ferrous heme irons in blood, the O-O bond is cleaved to create reactive oxygen species (ROS) on either the first or second oxygen according to the Fenton reaction.^23^ Then, the ROS overcome the parasite's antioxidant defense system and kill the malarial parasites.^23^ The pathway of ART to the products with antimalarial activity is briefly summarized in Figure 1A. This mechanism is applied similarly to other derivatives of ART that have the same peroxide groups. With this as an inspiration, an assay to detect ROS has been developed using a luminol reaction, an existing reaction used to detect blood in forensic science. In this reaction, luminol reacts with hydrogen peroxide under alkaline condition in the presence of catalyst hematin in blood to form a chemical compound that emits chemiluminescent signals at 425 nm that can be captured by an imaging system (Figure 1B).^25^

To mimic the mechanism of action of ART and the luminol reaction, the hydrogen peroxide solution is replaced with the drug solution. The hematin is used as the iron source to catalyze the reaction. The amount of peroxide group in ART and its derivatives correlates to the concentration of ART in the solution. It can be quantified using luminol and hematin in the sodium hydroxide solution.^26^ Before the probe can be used to test drug samples, a series of optimization and validation tests (the limit of detection/quantification (LOD/LOQ), specificity, repeatability, and robustness) was performed using the spectrophotometer. To make the platform field deployable, a microfluidic chip was designed to carry the mixing of the probe and API and their luminescent reaction.

### Optimization of the probe conditions.

#### The ratio of probe to API.

To determine the optimal concentration of hematin and luminol in 0.1 M NaOH (Sigma, St. Louis, MO), 10 mg each of hematin and luminol were dissolved in 50 mL of 0.1 M NaOH. The stock solution was serial diluted in phosphate-buffered saline (PBS; Sigma) to seven different concentrations (8, 6, 4, 2, 1, 0.5, 0.25 mg/50 mL). Different concentrations of hematin and luminol were tested with the solution of ART, ATS, and DHA (Sigma) 1 mg/mL. Forty microliters of the probe at different concentrations of luminol and hematin were mixed with 40 μL of ART, ATS, or DHA in a 96-well plate (Fisher, Pittsburgh, PA). Measurements were made immediately, after mixing in a 96-well plate, in the spectrophotometer (Molecular Devices, Sunnyvale, CA) with a kinetic mode of 5 minutes with 1-minute intervals at 425 nm emission wavelength. The measurements were repeated four times for each concentration of luminol and hematin.

#### The pH of the probe solution.

To find the optimal pH for the maximum luminescent signal, the solution of 1 M NaOH was diluted into four different concentrations (0.5, 0.1, 0.05, 0.01 M) corresponding to four different pH values. For the probe preparation, 50 mL solution at each concentration was mixed with 2 mg each of hematin and luminol. The concentrations of ART, ATS, and DHA for testing were 1 mg/mL. Measurements were made immediately, after mixing in a 96-well plate, in the spectrophotometer with a kinetic mode of 5 minutes with 1-minute intervals at 425 nm emission wavelength. The measurements were repeated four times for each concentration of NaOH.

#### The linear detection ranges of ART, ATS, and DHA.

To determine the linear detection range of the antimalarial probe, the standards from ART, ATS, and DHA were dissolved in ethanol (Fisher) at concentrations 6, 2, 1.6 mg/mL, respectively, and diluted into 11 concentrations to determine the LOD of each pharmaceutical and the linear range of the signal. The measurement was taken by the spectrophotometer for 20 minutes with 1-minute intervals using the 96-well plates. The peak signals were correlated to the concentration of API. The measurements were repeated at least twice for each concentration of ATS, ART, and DHA.

### Validation of the assay.

To validate the performance of the detection probe, specificity, repeatability, robustness tests were conducted.^27^ In all the validation tests, the probe solutions were prepared by adding 2 mg each of hematin and luminol into 50 mL of 0.1 M NaOH. All the measurements were taken using the spectrophotometer in the kinetic mode of 4 minutes with 1-minute intervals. The peak signals were observed at 2 minutes. The list of excipients from the Arsuamoon tablets was obtained for the specificity test.^28^ The detailed protocols of all the validation tests are included in Supplemental Materials section.

### Chip design and fabrication.

#### Design.

**Figure 2.**
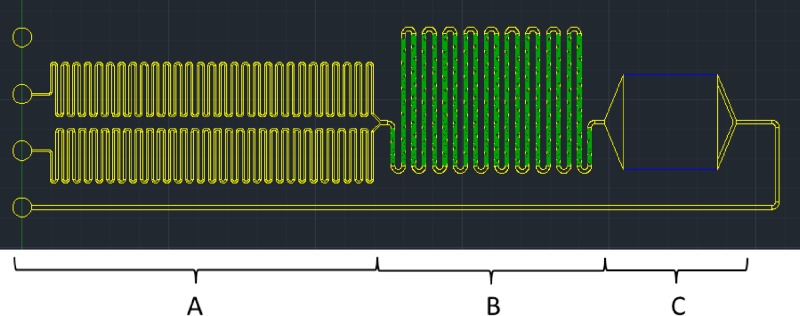
The computer-aided design (CAD) draw of chip (Autodesk, Inc., Waltham, MA) with the pressure drop channel (200 μm thick and 100 μm wide) (**A**), the mixing channel (200 μm thick and 200 μm wide) with herringbone patterns (40 μm thick and 50 μm wide) (**B**), and the reaction chamber (5 by 5 and 3 mm thick) (**C**).

For the luminescent detection, the signal was based on a chemical reaction of the probe with API. The signal strength correlated with the volume of the probe and the API. To be able to detect the luminescent light, the chip design contained two inlets for probe and API that injected the solutions through two long independent channels that were used to increase the pressure drop (Figure 2A) and then combined together into a single channel with multiple patterns of herringbone structure for mixing (Figure 2B). The herringbone patterns were the passive mixing methods in microfluidic channel where the Reynolds number was less than 1.^29^ After the mixing region, the solution entered a reaction chamber where the API and probe react to emit luminescent light captured by an imaging system (Figure 2C).

#### Fabrication.

The mold contained three layers of different heights made from SU-8 (MicroChem, Westborough, MA) and aluminum (McMaster-Carr, Robbinsville, NJ) on silicon wafer. The first layer was the 200 μm channel made from SU-8 2100 photoresist on a 10-in. silicon wafer following the protocol from MicroChem until the post-exposure bake. After that, the second layer of SU-8 2025 photoresist was spun on top of the first layer. The mold was developed in SU-8 developer. The third layer was made from aluminium with the dimension 5 × 5 × 3 mm. These blocks were glued to SU-8 channel using super glues. After that, the mold was silanized with trichlorosilane (1H, 1H, 2H, 2H-perfluorooctyl) (Sigma) for 2 hours under vacuum. The chips were made from PDMS (Fisher) with 10:1 ratio of base and curing agent for 3 hours at 85°C. The PDMS slabs were bonded to a glass slide using oxygen plasma treatment for 1 minute, then 3 hours at 85°C, and at room temperature overnight before ready to use.^30^

#### Complete dissolution of the pill and testing on chip.

The probe was prepared as described above. Five different tablets of Arsuamoon (50 mg of ATS/tablet) was weighed and crushed. The amount of powder that was equal to the weight of one tablet was dissolved in 62.5 mL of PBS to achieve 0.8 mg/mL concentration of ATS. Three samples were prepared. The mixture was stirred for 30 minutes and filtered through hydrophilic polypropylene membranes with 0.45 μm pores (Pall Corporation, Port Washington, NY). In parallel, a standard at 0.8 mg/mL of pure ATS was prepared as the control to compare with the tablet samples. The measurements were repeated five times for the same standard solution and one time for each tablet sample.

To determine the accuracy of the method, we compare our assay's performance with HPLC, the gold standard method for pharmaceutical analysis. The HPLC results were conducted at United States Pharmacopeia (USP) facility in Rockville, MD. There were three solvents used for the mobile phase: water (pH adjusted to 3 by formic acid), acetonitrile, and methanol with 8:11:1 ratio.^31^ The C18 reverse phase column (250 × 4.6 mm, particle size 5 μm) was used (Agilent, Santa Clara, CA). The ATS standard was prepared at the concentration of 4 mg/mL in acetonitrile. For the tablets, five tablets from the same batch were weighed and crushed into powder using the paddle. The amount of powder according to the weight of one tablet was measured and dissolved in acetonitrile. The solution was then filtered through a 0.2-μm hydrophilic polypropylene syringe filter. For the HPLC operation, the flow rate of the samples was 0.8 mL/min. The UV detection wavelength was at 216 nm. The injection volume was 20 μL. The total run time was 15 minutes.

## Results

This section shows our results in probe optimization, the linear detection range of the probe, and the validation of the probe's performance and the results of our validation scheme on the chips using both pure ATS sample and Arsuamoon tablets collected by USP from the field.

### Optimization of the probe conditions.

**Figure 3.**
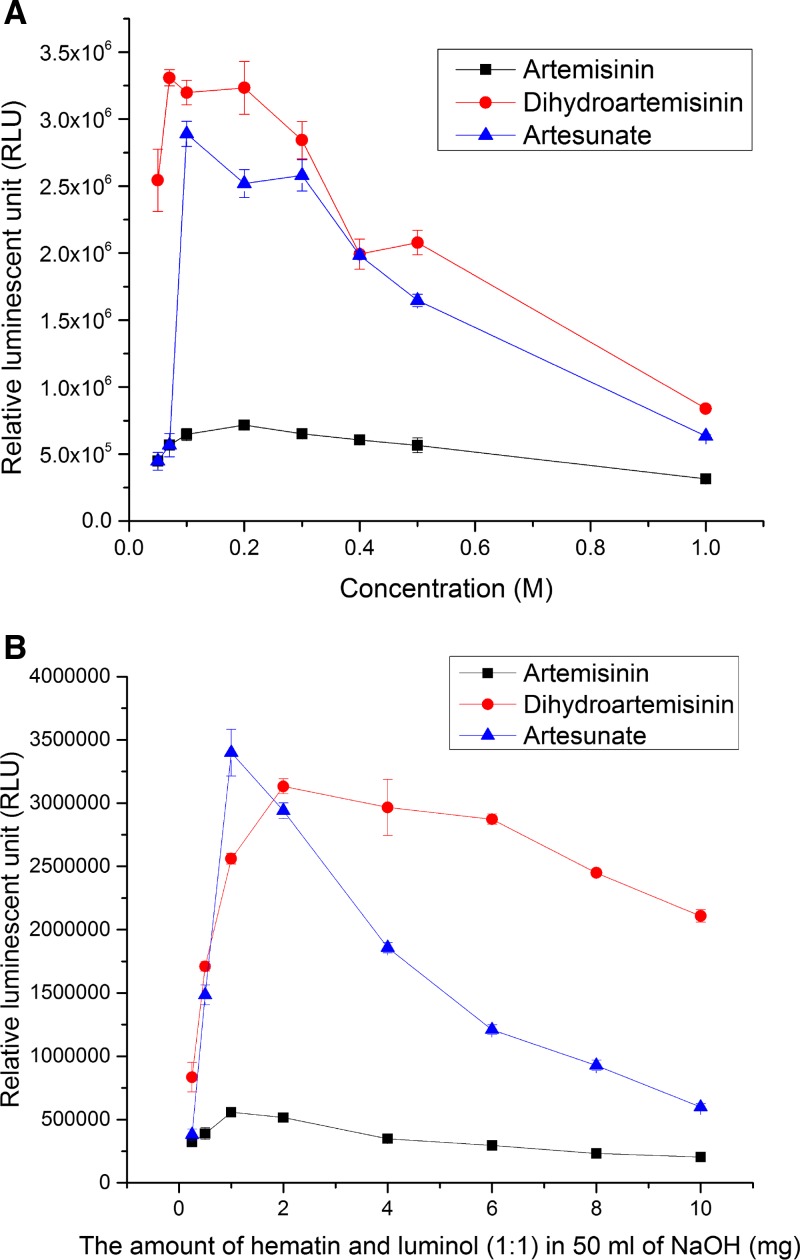
(**A**) The effect of different concentrations of sodium hydroxide on the luminescent signal from artemisinin (ART), artesunate (ATS), and dihydroartemisinin (DHA) (*N* = 4). (**B**) The effect of different ratio of probe to API on the luminescent signal of ART, ATS, and DHA (*N* = 4). The experiments were conducted in a 96-well plate system.

The results of probe optimization are shown in Figure 3. Figure 3A shows the curves of luminescent signals of ART, ATS, and DHA. The curve peaked at 0.1 M NaOH for ATS. For DHA and ART, the maximum signals were obtained at 0.07 and 0.2 M, respectively. The standard deviations were relatively small (less than 3%) at their peak values. The ratio of hematin and luminol to API curves showed that 2 mg each of hematin and luminol in 50 mL of 0.1 M NaOH M gave the highest signal for ART and DHA. For ATS, at 1 mg of hematin and luminol, the signal was the strongest, but only 10% more than 2 mg measurement and the standard deviation was significantly larger than the others (Figure 3B).

### The linear detection ranges of ART, ATS, and DHA.

From the plots in Supplemental Figure 1, the linear ranges of ART, ATS, and DHA using the luminol and hematin as the detection probe were determined as 0.1–1.5, 0.1–1.6, and 0.1–0.8 mg/mL, respectively.

### Validation of system.

#### Specificity.

**Figure 4.**
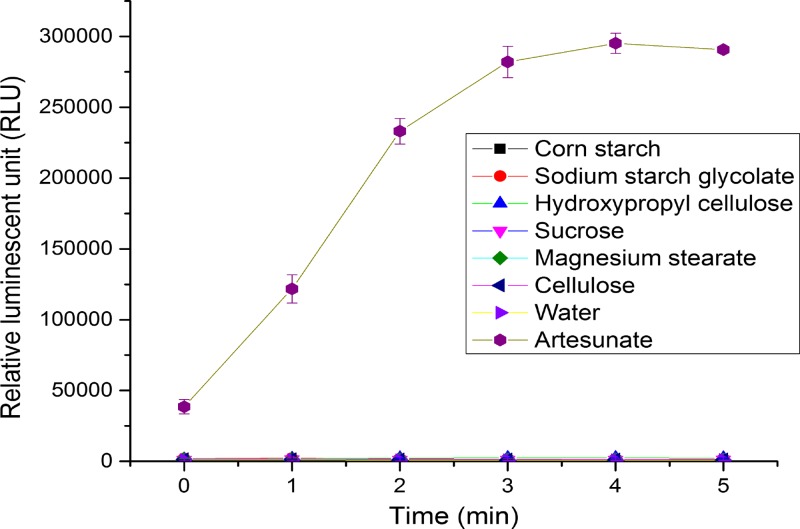
The graph of the luminescent signal of six different excipients in the Arsuamoon tablets that were measured separately with the probe vs. time (*N* = 5).^28^ The concentration of each analyte was 5 mg/mL. The detection wavelength is 425 nm and the experimental time is 5 minutes with 1-minute intervals. The experiments were conducted in a 96-well plate system.

The excipients (corn starch, sodium starch glycolate, hydroxypropyl cellulose, sucrose, magnesium stearate, and cellulose) were mixed with the probes and the signals were measured over time. From Figure 4, the signals were similar to the signal of the blank that was water or ethanol over 5 minutes.

#### Repeatability.

**Figure 5.**
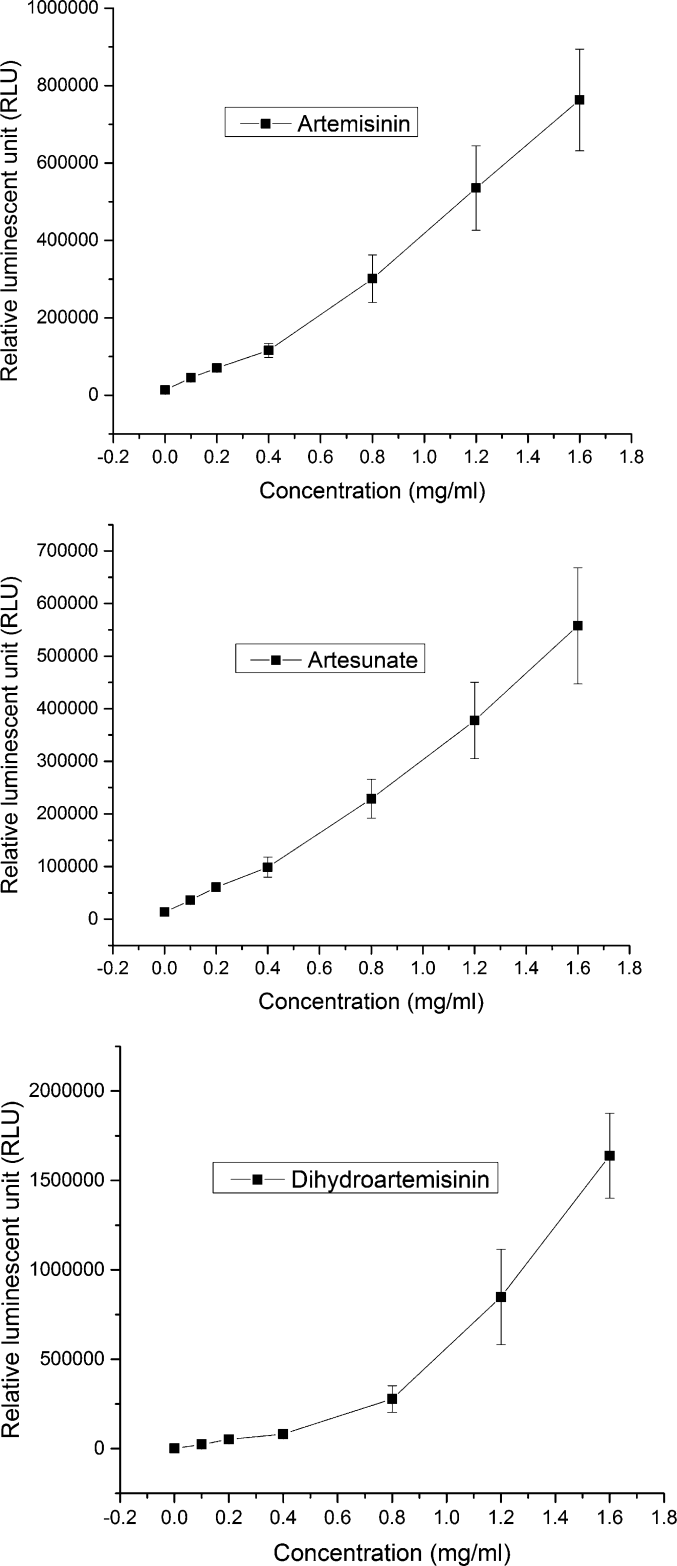
The graphs of the luminescent signal vs. concentration for the repeatability tests for artemisinin (ART), artesunate (ATS), and dihydroartemisinin (DHA) over seven different concentrations: 1.6, 1.2, 0.8, 0.4, 0.2, 0.1 mg/mL for ART, ATS, and DHA (*N* = 6). The experiments were conducted in a 96-well plate system.

Figure 5 shows the plots of repeatability tests of ART, ATS, and DHA for seven different concentrations. The ranges of the concentrations were picked based on the linear range of the signals versus the concentrations. The ranges were 0.1–1.6 mg/mL for ART and ATS, and 0.05–0.8 mg/mL for DHA.

#### Robustness.

**Figure 6.**
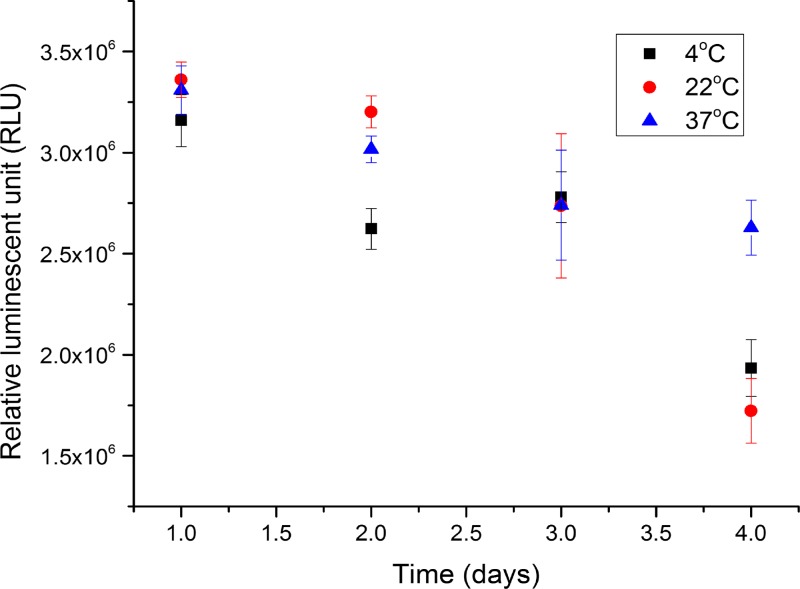
The graph of luminescent signal over time (days) for the probes stored at three different 4, 22, and 37°C with artesunate (ATS) using the spectrophotometer every day for 4 days including error bar (*N* = 8). The experiments were conducted in a 96-well plate system.

As shown in Figure 6, on the first day, there was no significant difference between three detection solutions at different temperatures on the first day, although the signals at 22 and 37°C are slightly more than the one at 4°C. After day 1, the signal from solution stored at 4°C decreases significantly (more than 20% on the second day). At 22°C, the signal decreases slightly on the second day, and more than 20% on the third day, and about 50% on the fourth day. For solution stored at 37°C, the signal decreases slightly over time and reaches ∼20% on the fourth day.

### Field sample testing on chip.

After the validation tests, we tested the antimalarial detection probe on chips using both pure standard ATS and ATS tablets from the field.

During the experimental set up, fluorescein was added to the probe solution to change the emission wavelength from 425 to 530 nm.^32^ The standard curve at four different concentrations (0.4, 0.8, 1.2, 1.6 mg/mL) was created on the chip. For each concentration, the chemiluminescent signal was plotted over time in Supplemental Figure 2A. The peak signals were captured for each concentration and plotted against the concentrations in Supplemental Figure 2B. The standard deviation was insignificant for all concentrations. Supplemental Figure 2B shows a linear relationship between the luminescent signal and the concentrations of ATS ranging from 0.4 to 1.6 mg/mL. The experimental set up for the ATS standard curve was included in the Supplemental Materials section.

### Arsuamoon field sample testing.

For quantification, a standard solution was prepared and five readings were taken for the same sample. The average signal is 2.38445 V with % relative standard deviation (RSD) of 5.75 (Table 1). For the three Arsuamoon samples, the average signal is 2.44801 V with %RSD of 11.4. Based on the standard, the percentage of recovery is 104% or 52 mg compared with 50 mg of ATS stated on the package (Table 2). The results from HPLC showed that the %recovery of ATS from the tablet is 93.4 (47.7 mg) with 1.7% RSD. The percentage difference between our assay and HPLC was 10.6.

## Discussion

The optimization tests for the probes showed that the luminescent signal depends on two different factors: the concentration of luminol and hematin and the pH of the solution determined by the concentration of sodium hydroxide. From Figure 3, the optimal condition to produce the highest luminescent signals was 2 mg each of hematin and luminol in 50 mL of 0.1 M NaOH. For the LOD/LOQ tests, the data in Table 1 show that the methods covered a wide range of concentrations of ART, ATS, and DHA from μg to mg range. The upper limit at 2 mg/mL helped to decrease the volume used to dissolve the 50 mg-ATS tablets down to 25 mL.

In the system validation, the specificity tests demonstrated that the probe was highly specific to only ATS (100% for LOQ range) in the tablets and could be used to quantitatively estimate the concentration of the ATS in the tablets. The repeatability tests showed that the signals for different concentrations were distinguished from each other with the standard deviations. Overall, the signals from antimalarial probe were reproducible for ATS, ART, and DHA over a range of concentrations 0.1–1.6 mg/mL. Finally, in the robustness tests, the reagent storage temperature did not affect the luminescent signal significantly on the first day. The signal from the solution stored at 4°C was lower on the second day because the solution was exposed to ambient light occasionally when the fridge was open. Therefore, the solution should be prepared freshly everyday at the beginning of the testing to ensure the consistent measurement from day to day.

To quantify the API content of ATS tablets, we developed an ATS standard curve on chip using photodiode as the detector. For the analysis, two different approaches, peak-picking and integration of signal under the curve methods from the plot of the luminescent signal over time, were chosen. By looking at the peak of the curves for four concentrations, we can distinguish and quantify different concentrations of ATS in the solution in the range 0.4–1.6 mg/mL. In Supplemental Figure 2A, each concentration had a unique peak value between 2- and 3-minutes interval. On the other hand, the integration of the signal under the curve for 4 minutes could not distinguish different concentrations because the graph of the relative luminescent signal over time (Supplemental Figure 2A) showed that the higher the concentration, the more rapid the signal decays, especially after 3 minutes. Thus, the peak signal obtained between 2 and 3 minutes of reaction time was picked to plot the standard curve of ATS on a chip (Supplemental Figure 2B). The voltage signal from Supplemental Figure 2 for 0.8 mg/mL was lower than the one from the standard solution in Table 1. This was due to the change in the position of the chip relative to the photodiode. To increase the number of photons collected by the photodiode, we moved the chip closer to it and thus increased the voltage signal linearly for all concentrations. This did not affect the linearity between the ATS concentrations and the luminescent signal in the range of 0.4–1.6 mg/mL. From Supplemental Figure 2B, the linear range of luminol reaction with ATS was in the range 0.4–1.6 mg/mL. Thus, we pick 0.8 mg/mL as the control standard to quantify the API content in Arsuamoon tablets. From Table 2, the percentage of recovery is 104%, which is 8.4% higher than HPLC results. Moreover, the %RSD of ours assay was significantly higher than HPLC (11.4% versus 0.7%). This may be due to the variations from chip to chip because each test was run on a new chip. The alignment of the reaction chamber on chip was done manually by hand, which can lead to the variation in the position of the chamber compared with the photodiode, which affects the amount of luminescent captured by the photodiode. The system still has many factors needed to be controlled and improved to minimize the variations between tests.

Overall, we demonstrated that our system was able to quantify the ATS tablets as well as the conventional 96-well plate in the spectrophotometer and obtain comparable results to the HPLC. Moreover, with our platform, the chips can be cleaned and reused up to three times before disposal. This reduces the chip manufacture cost per test to about $0.5, which is higher than the cost of the wells in the plate ($0.05/well or $4.8/plate). However, the cost per chip can be reduced by using plastics as the material for chip's production (estimated to be less than $0.1/chip). Besides that, our detection platform, including the pressure system and a photodiode, costs less than $3,000 compared with the spectrophotometer (> $40,000) or HPLC (up to $100,000).^4^,^33^ We have tried to capture signal using cell phone camera, but it is not sensitive enough to distinguish different concentrations of ATS. In addition, our system can potentially be miniaturized into a compact suitcase for field testing whereas the spectrophotometer and HPLC have to be set up in advanced laboratory with annual maintenance. The drawback of our technology is that the variation from reading to reading is significantly higher than that of HPLC. This can be reduced by exploring different chip fabrication methods such as injection molding and hot embossing. Our next step is to modify the current system into field-deployable platform and test its performance with different ATS field samples in both single and fixed dose tablets.

## Conclusion

Substandard pharmaceuticals have continued to be a major problem that affects many countries all over the world, especially in Africa and southeast Asia. Current technologies provide good tools to help alleviate the problem, but there is still a gap in technology to fill in. To address these gaps, we have developed an assay to detect substandard antimalarial tablets. The assay includes two components: chip design and probe development. In the probe development, we have developed and characterized the detection probes for ART, ATS, and DHA. In conjunction to probe optimization, we designed a novel microfluidic chip to test the field samples of Arsuamoon tablets. Despite the promise of our technology and its potential impact, we recognize that more optimization is needed along with integration into a fully automated field-ready system, which is currently underway. Our ultimate goal is to build and validate the performance of an affordable, easy to use, sensitive, robust, and portable platform that does real time quantification of antimalarials using luminescent signal in resource-limited settings with high specificity and sensitivity. We believe our current results are the first step in that direction.

## Supplementary Material

Supplemental Materials.

## Figures and Tables

**Table 1:** The summary of the measurements for the artesunate (ATS) standard on microfluidic chip

Sample name	Info	Theoretical concentration (mg/mL)	Maximum voltage	Average reading	%RSD
Standard	Reading 1	0.8	2.5536	2.38448	5.75
	Reading 2	0.8	2.25459		
	Reading 3	0.8	2.45197		
	Reading 4	0.8	2.23241		
	Reading 5	0.8	2.42984		

Five readings were conducted. The average signal was 2.38448 V with an RSD of 5.75%.

**Table 2:** The summary of the measurements of three different samples of Arsuamoon tablets

Sample name	Info	Theoretical concentration (mg/mL)	Maximum voltage	%Recovery	%RSD
Standard	Average reading	0.8	2.38448	100	5.75
Arsuamoon	Sample preparation 1	0.8	2.32589	99.1	−
	Sample preparation 2	0.8	2.76852	117.95	−
	Sample preparation 3	0.8	2.24963	95.85	−
	Average	0.8	2.44801	104.3	11.4
	HPLC	4	−	95.4	0.7

The average signal was 2.44801 V with an RSD of 11.4%. The average percentage of recovery was 104.3. The percentage of recovery from high-performance liquid chromatography (HPLC) was 95.4 with a %RSD of 0.7.
