# Supplementary material for: Rapid and Specific Drug Quality Testing Assay for Artemisinin and Its Derivatives Using a Luminescent Reaction and Novel Microfluidic Technology
Source: Am J Trop Med Hyg. 2015 Jun 3;92(Suppl 6):24–30. doi: 10.4269/ajtmh.14-0392 (PMC4455072; doi:10.4269/ajtmh.14-0392)
Supplement: Supplementary file 1 [file SD2.pdf]

Supplemental Materials

THE LINEAR DETECTION RANGES OF ART, ATS, AND DHA

Validation of the assay. *Specificity.* A list of excipients from an Arsuamoon tablet was obtained from WHO and United States Pharmacopeia (USP) documents: corn starch, sodium starch glycolate, hydroxypropyl cellulose, sucrose, magnesium stearate, and cellulose.[28] Five milligrams of each excipient were dissolved in 1 mL of distilled water. Thirty microliters of excipient solutions were mixed with 30 μL of probe. The signals were captured using the spectrophotometer for 5 minutes with 1-minute intervals. The measurements were repeated five times for each excipient.

Repeatability. The stock solution of each pharmaceutical (2 mg/mL for artesunate (ATS) and artemisinin (ART); 1 mg/mL for dihydroartemisinin [DHA]) in ethanol was identified as 100% and diluted into seven other different concentrations at 80%, 60%, 40%, 20%, 10%, 5%, and 0% compared with the stock solutions. Five sets of the standards from the same stock solutions were prepared. Thirty microliters of active pharmaceutical ingredient (API) solution and probe were mixed in a 96-well plate. The measurements were taken immediately using the spectrophotometer and repeated six times for each concentration of API.

Robustness. Three 50 mL solutions of the probe were made separately and stored at three different temperatures 4, 22, 37°C for 3 hours before the first measurements. The ATS samples were prepared freshly every day by dissolving 2 mg in 200 μL of ethanol and diluting in 1.9 mL of water at the temperature according to the probe solution's temperature. For the detection, 40 μL of ATS and 40 μL of the probe were mixed in a 96-well plate. The measurements were taken for each temperature ($N = 8$) every day until the signal started to decrease more than 20% compared with the previous measurement in all temperatures.

Experimental set up for on chip testing. A standard curve of ATS in phosphate buffered saline (PBS) was developed using photodiode as the sensor in optical nest. To prepare the probe solution, 2 mg each of luminol, hematin, and fluorescein (Sigma) was dissolved in 50 mL of 0.1 M NaOH. Fluorescein was added to alter the emission wavelength of the luminescent signal from 425 to 530 nm. The luminescent light from the luminol reaction with ATS acts as the transferred energy source to excite fluorescein and then the fluorescein emitted the signal at 530 nm.[31] For the API, 4 mg of API was weighed and dissolved in 200 μL of ethanol 200 proof by vortex and diluted in 1.8 mL of PBS to achieve the 2 mg/mL concentration. The solution was later diluted in 500 μL of PBS to bring the concentration down to 1.6 mg/mL. Then, a serial dilution from 1.6 to 1.2, 0.8, 0.4 mg/mL was made by adding 750, 500, 250 μL of 1.6 mg/mL solution into 250, 500, 750 μL of PBS in 2-mL tubes labeled with concentrations and mixing by vortex.

The pressure was set to 5 psi. The signals were captured for 5 minutes at 16 Hz. The peak signal analysis was used to create a standard curve with four different concentrations. The measurements were repeated three times for each concentration. The chips were reused for three times. Between measurements, the chips were washed with water for 3 minutes and air-dried for 2 minutes. To determine the cleaning protocol, we experimented with different washing (3, 4, 5 minutes) and air-dry (1, 2, 3 minutes) times between the measurements. After washing the chips, we flowed the pure ATS solution in the API inlet and water in the probe inlet and captured the signal over 5 minutes. The signals were the same as the background signal (data not shown). Based on the results, we concluded that the chip required to be washed for 3 minutes and dried for 2 minutes between measurements.

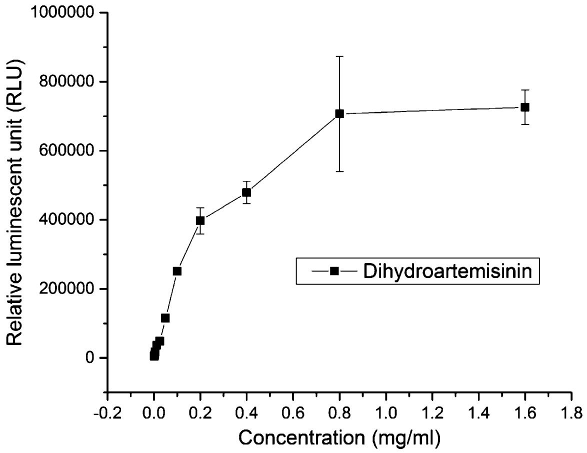

Supplemental Figure 1. The LOD/LOQ of ART, ATS, and DHA. Each graph represents the relative luminescent units vs. the concentration of active pharmaceutical ingredient (API) ($N = 2$).

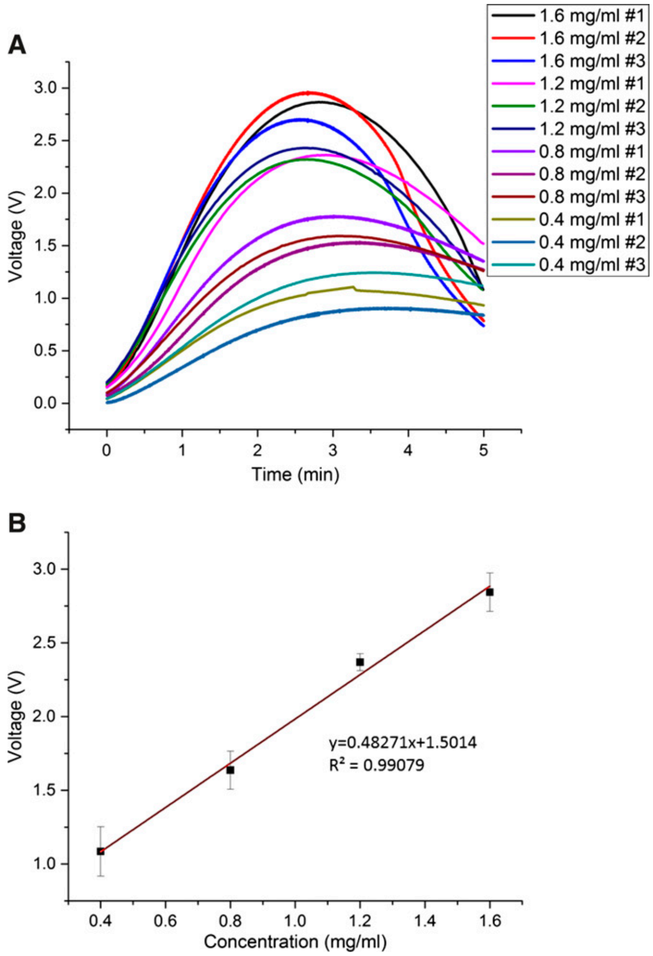

Supplemental Figure 2. (**A**) The luminescent signal recorded in voltage over time for each concentration (four different concentrations: 1.6, 1.2, 0.8, 0.4 mg/mL). The measurements were taken at 16 Hz for 5 minutes ($N = 3$). (**B**) The graph of voltage vs. the four different concentrations of ATS on chip. The experiments were conducted in microfluidic chips.
